# The evolutionary scope and neurological disease linkage of yeast-prion-like proteins in humans

**DOI:** 10.1186/s13062-016-0134-5

**Published:** 2016-07-26

**Authors:** Lu An, Paul M. Harrison

**Affiliations:** Department of Biology, McGill University, Montreal, QC Canada

**Keywords:** Prion, Amyloid, Yeast, Human, Disease, Neurodegenerative, Poly-Q expansion, Evolution, Glutamine, Asparagine

## Abstract

**Background:**

Prions are proteinaceous particles that propagate alternative protein conformations/states to further copies of the same proteins, and are transmitted from cell-to-cell, and organism-to-organism. Prions are usually made of the beta-sheet rich assemblies termed amyloid. The original prion protein PrP causes devastating neurodegenerative disorders in humans and other mammals. In the yeast *Saccharomyces cerevisiae*, many prion-forming proteins have been observed; a prominent feature of these proteins is an intrinsically disordered domain rich in glutamine (Q) and asparagine (N) residues. Several human proteins that are yeast-prion-like, in particular those with poly-glutamine (poly-Q) expansions, have been experimentally implicated in human neurodegenerative diseases.

**Results:**

Here, we have constructed a comprehensive list of human yeast-prion-like proteins that are linked to human neurological disease. Surprisingly, different methods to annotate yeast-prion-like proteins in humans have limited intersection. However, independent of annotation method, we find that human yeast-prion-like proteins as a group have a statistically significant genetic linkage to neurological disease, that is caused specifically by linkage to neurodegenerative diseases. This is despite: (i) no especially high expression of yeast-prion-like proteins in the central nervous system, or (ii) no general enrichment of intrinsically disordered proteins in neurological/neurodegenerative diseases. Cytoskeletal proteins are significantly overrepresented in the set of human yeast-prion-like neurological proteins. Whether involved in neurological pathomechanisms or not, yeast-prion-like proteins in humans have very limited conservation outside of *Deuterostomia* (< ~10 %) with only a handful having prion-like character in both human and *S. cerevisiae*. The only such protein with a disease linkage is PUB1/TIA1, which functions as a stress granule component. Thus, the yeast-prion-like character of proteins linked to neurodegenerative diseases has not been conserved over the deep evolutionary time since the last common ancestor of yeasts and humans.

**Conclusion:**

Our results provide a comprehensive picture of yeast-prion-like proteins in humans and contribute to the strategic basis for experimental investigation of the link between yeast-prion-like protein character and neurological disease.

**Reviewers:**

Reviewed by Istvan Simon and Alexander Schleiffer. For the full reviews, please go to the Reviewers’ comments section.

**Electronic supplementary material:**

The online version of this article (doi:10.1186/s13062-016-0134-5) contains supplementary material, which is available to authorized users.

## Background

Prions were originally identified as proteinaceous infectious particles that caused devastating neurological disorders in humans and other mammals. These prions are particles that propagate alternative states of proteins, by co-opting further copies of the same proteins. In the yeast *Saccharomyces cerevisiae*, these alternative states can be transmitted sustainably during budding, mating or laboratory infection protocols. Yeast prions propagate heritable phenotypes, uncover hidden genetic variation, function in large-scale gene regulation, and can act like diseases. The first well-characterized yeast prions, that underlie the [PSI+] and [URE3] prion states, are propagating amyloids (*i.e.*, fibrillar beta-sheet aggregates) of the proteins Sup35p and Ure2p. The protein Sup35p functions as part of the translation termination complex. Formation of [PSI+] prions reduces the efficiency of translation termination and increases levels of nonsense-codon read-through [1, 2]. Such read-through has been shown to be a potential mechanism to uncover cryptic genetic variation [3, 4]. [URE3] causes upregulation of poor nitrogen source usage, even when rich sources are available [5–7]. Prion variants may be considered as diseases of *S. cerevisiae* in some contexts [8, 9]. A more recently discovered example, the [MOT3+] prion, has been shown to function in controlling the acquisition of multicellularity in *S. cerevisiae* [10]. There are now at least 10 known prions of *S. cerevisiae* that are propagated by amyloids [11, 12].

A common compositional feature of almost all amyloid-based yeast prions is bias for asparagine (N) and/or glutamine (Q) residues [12, 13]. These yeast prions are also amongst the proteins with the highest degrees of intrinsic disorder in the yeast proteome [11]. Bioinformatic surveys have revealed the existence of hundreds of proteins with such N/Q-richness in *S. cerevisiae* and diverse other fungi [14–16]. Evolutionary analysis showed that the [PSI+] prion N/Q bias is conserved across fungal clades that diverged >1 billion years ago, with only eight other *S. cerevisiae* proteins showing similar, phylogenetically deep patterns of conservation of yeast-prion-like character [15]. A large population of yeast-prion-like proteins emerged early in the evolution of the budding yeast evolutionary class *Saccharomycetes*, as a result of mutational trends that lead to the formation of more polyasparagine runs, thus providing an evolutionary ‘test set’ out of which several prion-forming domains have arisen [17].

Prion-like propagation of aggregates between neurons has been demonstrated in several neurodegenerative diseases, including amyotophic lateral sclerosis (ALS) [18, 19], Alzheimer’s disease [20, 21] and Parkinson’s disease [22, 23]. Several human proteins have prion-like N/Q-rich domains that have been experimentally linked to neurodegenerative pathomechanisms. Cytoplasmic aggregates of the RNA-binding protein FUS, which contains a Q-rich domain, are implicated in ALS, and its aggregation has been re-capitulated in an induced *S. cerevisiae* proteinopathy [24]. Mutations in two yeast-prion-like proteins hnRNPA2B1 and hnRNPA1 initiate neurodegenerative disease in humans through amyloid formation [25]. Also, pathogenic proteins in at least nine other neurodegenerative disorders, such as Huntington’s disease, have disease-linked poly-Q expansions.

Here, we derive a comprehensive list of yeast-prion-like proteins for humans using three different methods and assess their evolution through comparison to a diverse panel of eukaryotes. We also characterize the linkage of yeast-prion-like proteins to neurological diseases, showing that they have a specific relationship to neurodegeneration/muscular degeneration that is not due to other more general factors, such as intrinsic disorder or high tissue-specifc expression. Human yeast-prion-like proteins are largely novel in evolution since the last common ancestor of *Deuterostomia*, with only one neurologically relevant protein (PUB1/TIA1) having yeast-prion-like character in both humans and yeast.

## Methods

### Annotation of proteomes

Complete eukaryotic proteomes for the organisms listed in Table 1 were downloaded from the NCBI genome website [26]. These were annotated for domains with prion-like N/Q-rich composition, using the LPS program [11, 27, 28], as described previously [17]. Prion-like domains were also predicted using the programs PAPA [29] and PLAAC [30]. With the latter, predictions were made using both the background amino-acid composition of budding yeast, and of each individual eukaryotic proteome. Intrinsically disordered proteins were annotated using the program IUPRED [31].

**Table 1:** Summary of the distribution of yeast-prion-like proteins in the eukaryotic domain

Species short name	Species binomial name *(some clade names are listed in this column for clarity)*	TOTALS OF YEAST-PRION-LIKE PROTEINS^a^			CONSERVATION OF PRION-LIKE PROTEINS FROM YEAST^b^			CONSERVATION OF YEAST-PRION-LIKE PROTEINS FROM HUMAN^b^		
		(1) TOTAL # of N/Q-rich proteins (NQPs)	(2) TOTAL # of prion predictions (yeast background)	(3) TOTAL # of prion predictions (organism’s background)	(4) # of YEAST proteins conserved as orthologs (total 5879)	(5)	(6)	(7) # of HUMAN proteins conserved as orthologs (total 101,933)	(8)	(9)
YEAST	Saccharomyces cerevisiae	***285*** ***(*** ***4*** **.** ***9*** ***%)***	***186*** ***(3*** **.** ***2*** ***%)***	***186*** ***(*** ***3*** ***.*** ***2*** ***%)***	--	--	--	4076 (4.0 %)	9 (0.7 %)	4
TRICHOPLAX	Trichoplax adhaerens	220 (1.9 %)	308 (2.7 %)	***276*** ***(*** ***2*** ***.*** ***4*** ***%)***	2223 (38 %)	3 (1.0 %)	5	9992 (9.8 %)	24 (1.9 %)	24
SPONGE	Amphimedon queenslandica	192 (1.4 %)	217 (1.6 %)	166 (1.2 %)	2230 (38 %)	1 (0.4 %)	5	9565 (9.4 %)	16 (1.3 %)	27
	**Protostomia**									
DROSOPHILA	Drosophila melanogaster	**3085** **(11** **%)**	**2064** **(** **7** **.** **7** **%)**	**1653** **(** **5** **.** **9** **%)**	2176 (37 %)	26 (9.1 %)	14	10138 (10 %)	117 (9.2 %)	72
DAPHNIA	Daphnia pulex	461 (1.5 %)	509 (1.7 %)	307 (1.0 %)	2209 (38 %)	5 (1.8 %)	4	10609 (10 %)	44 (3.5 %)	30
C_ELEGANS	Caenorhabditis elegans	827 (2.6 %)	***1112*** ***(*** ***3*** ***.*** ***5*** ***%)***	732 (2.3 %)	2044 (35 %)	11 (3.9 %)	14	8670 (8.5 %)	41 (3.2 %)	27
OYSTER	Crassostrea gigas	1059 (2.3 %)	1414 (3.1 %)	988 (2.1 %)	2333 (40 %)	7 (2.5 %)	14	12934 (13 %)	126 (9.9 %)	89
APLYSIA	Aplysia californica	***1342*** ***(*** ***4*** ***.*** ***9*** ***%)***	***954*** ***(*** ***3*** ***.*** ***5*** ***%)***	***645*** ***(*** ***2*** ***.*** ***4*** ***%)***	2310 (39 %)	14 (4.9 %)	12	11017 (11 %)	35 (2.8 %)	30
LEECH	Helobdella robusta	**3593** **(** **15** **%)**	**2053** **(** **8** . **8** **%)**	**1605** **(** **6** **.** **7** **%)**	2159 (37 %)	13 (4.6 %)	7	10834 (11 %)	65 (5.1 %)	30
	**Deuterostomia**									
SEA_URCHIN	Strongylocentrotus purpuratus	1007 (2.8 %)	909 (2.5 %)	581 (1.6 %)	2355 (40 %)	12 (4.2 %)	13	13596 (13 %)	117 (9.2 %)	59
ZEBRAFISH	Danio rerio	671 (1.6 %)	805 (1.9 %)	617 (1.5 %)	2389 (41 %)	5 (1.8 %)	6	20283 (20 %)	213 (17 %)	143
FUGU	Takifugu rubripes	940 (2.0 %)	912 (1.9 %)	719 (1.5 %)	2314 (39 %)	4 (1.4 %)	4	20340 (20 %)	205 (17 %)	129
CHICKEN	Gallus gallus	276 (1.7 %)	227 (1.4 %)	178 (1.1 %)	2176 (37 %)	8 (2.8 %)	3	18441 (18 %)	247 (19 %)	144
ANOLIS	Anolis caroliniensis	798 (2.5 %)	624 (1.9 %)	468 (1.5 %)	2311 (39 %)	5 (1.8 %)	3	22209 (22 %)	320 (25 %)	197
MOUSE	Mus musculus	1001 (1.8 %)	675 (1.2 %)	497 (0.9 %)	2397 (41 %)	6 (2 %)	5	30987 (30 %)	514 (41 %)	314
HUMAN	Homo sapiens	1269 (1.2 %)	1012 (1.0 %)	766 (0.7 %)	2399 (41 %)	5 (1.8 %)	3	--	--	--
OPOSSUM	Monodelphis domestica	382 (1.7 %)	298 (1.4 %)	225 (1.0 %)	2329 (40 %)	7 (2.5 %)	3	22205 (22 %)	290 (23 %)	197
XENOPUS	Xenopus laevis	348 (1.5 %)	376 (1.7 %)	317 (1.4 %)	2299 (39 %)	9 (3.2 %)	4	19444 (19 %)	226 (18 %)	131
LATIMERIA	Latimeria calumnae	407 (1.7 %)	415 (1.8 %)	330 (1.4 %)	2276 (39 %)	7 (2.5 %)	2	19756 (19 %)	273 (22 %)	145
CIONA	Ciona intestinalis	283 (1.8 %)	491 (3.2 %)	***389*** ***(*** ***2*** ***.*** ***5*** ***%)***	2213 (38 %)	4 (1.4 %)	5	11547 (11 %)	72 (5.7 %)	67
HYDRA	Hydra vulgaris	712 (3.2 %)	489 (2.2 %)	431 (1.9 %)	2235 (38 %)	14 (4.9 %)	11	10605 (10 %)	41 (3.2 %)	36
CHOANAFLAGELLATE	Monosiga brevicollis	273 (3.0 %)	134 (1.5 %)	85 (0.9 %)	2015 (34 %)	4 (1.4 %)	5	6632 (6.5 %)	12 (0.9 %)	7
DICTYOSTELIUM	Dictyostelium discoideum	**5345** **(** **40** **%)**	**3067** **(** **23** **%)**	**2567** **(** **19** **%)**	2199 (37 %)	65 (23 %)	34	5839 (5.8 %)	37 (2.9 %)	31
PLASMODIUM	Plasmodium falciparum	**2649** **(** **50** **%)**	**1248** **(** **23** **%)**	**1143** **(** **22** **%)**	1351 (23 %)	23 (8.1 %)	20	3207 (3.1 %)	30 (2.4 %)	25
CHONDRUS	Chondrus crispus	49 (0.5 %)	93 (0.9 %)	62 (0.6 %)	1716 (29 %)	6 (2 %)	7	3790 (3.7 %)	2 (0.2 %)	5
	**Plants**									
PHYSCOMITRELLA	Physcomitrella patens	169 (0.5 %)	323 (0.9 %)	246 (0.7 %)	2279 (39 %)	5 (1.8 %)	3	6450 (6.3 %)	6 (0.5 %)	5
SELAGINELLA	Selaginella moellendorffii	285 (0.8 %)	243 (0.7 %)	164 (0.5 %)	2247 (38 %)	1 (0.4 %)	1	6370 (6.3 %)	10 (0.8 %)	4
AMBORELLA	Amborella trichopoda	171 (0.8 %)	251 (1.2 %)	164 (0.8 %)	2288 (39 %)	4 (1.4 %)	4	6079 (6.0 %)	13 (1.0 %)	4
BANANA	Musa acuminata	421 (1.0 %)	403 (1.0 %)	295 (0.7 %)	2281 (39 %)	7 (2.5 %)	4	6328 (6.2 %)	5 (0.4 %)	4
RICE	Oryza sativa	1118 (1.6 %)	928 (1.4 %)	540 (0.8 %)	2264 (39 %)	6 (2 %)	8	6144 (6.0 %)	9 (0.7 %)	8
ARABIDOPSIS	Arabidopsis thaliana	342 (1.0 %)	570 (1.7 %)	412 (1.2 %)	2300 (39 %)	7 (2.5 %)	7	6235 (6.2 %)	11 (0.9 %)	9
CHLAMYDOMONAS	Chlamydomonas reinhardtii	441 (3.0 %)	180 (1.2 %)	142 (1.0 %)	1914 (33 %)	1 (0.4 %)	0	5235 (5.2 %)	7 (0.6 %)	2
TRYPANOSOMA	Trypanosoma cruzi	359 (1.8 %)	225 (1.1 %)	172 (0.9 %)	1539 (26 %)	8 (2.8 %)	3	4032 (3.9 %)	9 (0.7 %)	13
GUILLARDIA	Guillardia theta	318 (1.3 %)	238 (1.0 %)	163 (0.7 %)	2115 (36 %)	4 (1.4 %)	4	6409 (6.3 %)	6 (0.5 %)	5

^a^The organisms are listed in the same order that they appear in the phylogenetic tree in Fig. 1. Organisms with percentages of NQPs in their proteomes >10 % are in bold underline text; those otherwise with >4 % NQPs in their proteomes are in bold italics. Where other organisms reach the level of the % prion predictions of these organisms, their totals of prion predictions are also given in bold italics. ‘Yeast background’ is the setting in the PLAAC program wherein the background composition in the yeast proteome is used for prion predictions, similarly for ‘Organism’s background’

^b^Columns (5), (6), (8) and (9) contain the following data

(5): # of YEAST N/Q-rich proteins conserved as N/Q-rich orthologs (total in YEAST =285), with the percentages this is of total YEAST NQPs in brackets

(6): # of YEAST prion predictions conserved as prion predictions (total in YEAST =186) (yeast background)

(8): # of HUMAN N/Q-rich proteins conserved as N/Q-rich orthologs (total in HUMAN =1269), with the percentages this is of total HUMAN NQPs in brackets

(9): # of HUMAN prion predictions conserved as prion predictions (total in HUMAN =1012) (yeast background)

### Evolutionary analysis

An organismal phylogeny of the eukaryotes was obtained from NCBI Taxonomy [32]. Organismal phylogenetic trees were drawn using phyloT [33] to generate a Newick format file, which was then input into Phylodendron [34]. Orthologs in the other eukaryotes for all of the human proteins were calculated using the bi-directional best hits method, as described previously [17].

### Other data sources

Lists of human genes/proteins linked genetically to diseases were obtained from OMIM [35]. A list of neurological diseases was obtained from the NINDS website [36], and a curated list of genes/proteins linked to neurodegeneration was obtained from UniProt [37]. These lists were cross-referenced with the OMIM data. Further genes linked to neurodegeneration/muscular degeneration were identified from curation of the scientific literature. The Gene Ontology database [38] was used to assess functional classifications.

Human tissue-specific gene expression data was obtained from the GXA Expression atlas Illumina body map [39] and from SpeCond [40]. To generate lists of tissue-specifically expressed genes from the Illumina body map, the genes had to be specifically expressed in the tissue in question above expression level cutoff of 0.5 in experiment EMTAB-513.

## Results and Discussion

### Conservation across the eukaryote domain

We consider yeast-prion-like proteins to be proteins with a domain that has yeast-prion-like character. This character is either a pronounced compositional bias for glutamine and/or asparagine residues as determined by the program LPS [11, 27, 28], or prediction of a prion domain by the programs PAPA or PLAAC [29, 30] (see Methods for details). The former are termed *N/Q-rich proteins* (NQPs), whereas the latter are termed *prion predictions*. In the human proteome, yeast-prion-like proteins are somewhat rarer than in *Saccharomyces cerevisiae* itself (occurring at a rate of 1-2 % versus 3-5 %; Table 1).

**Fig. 1:**
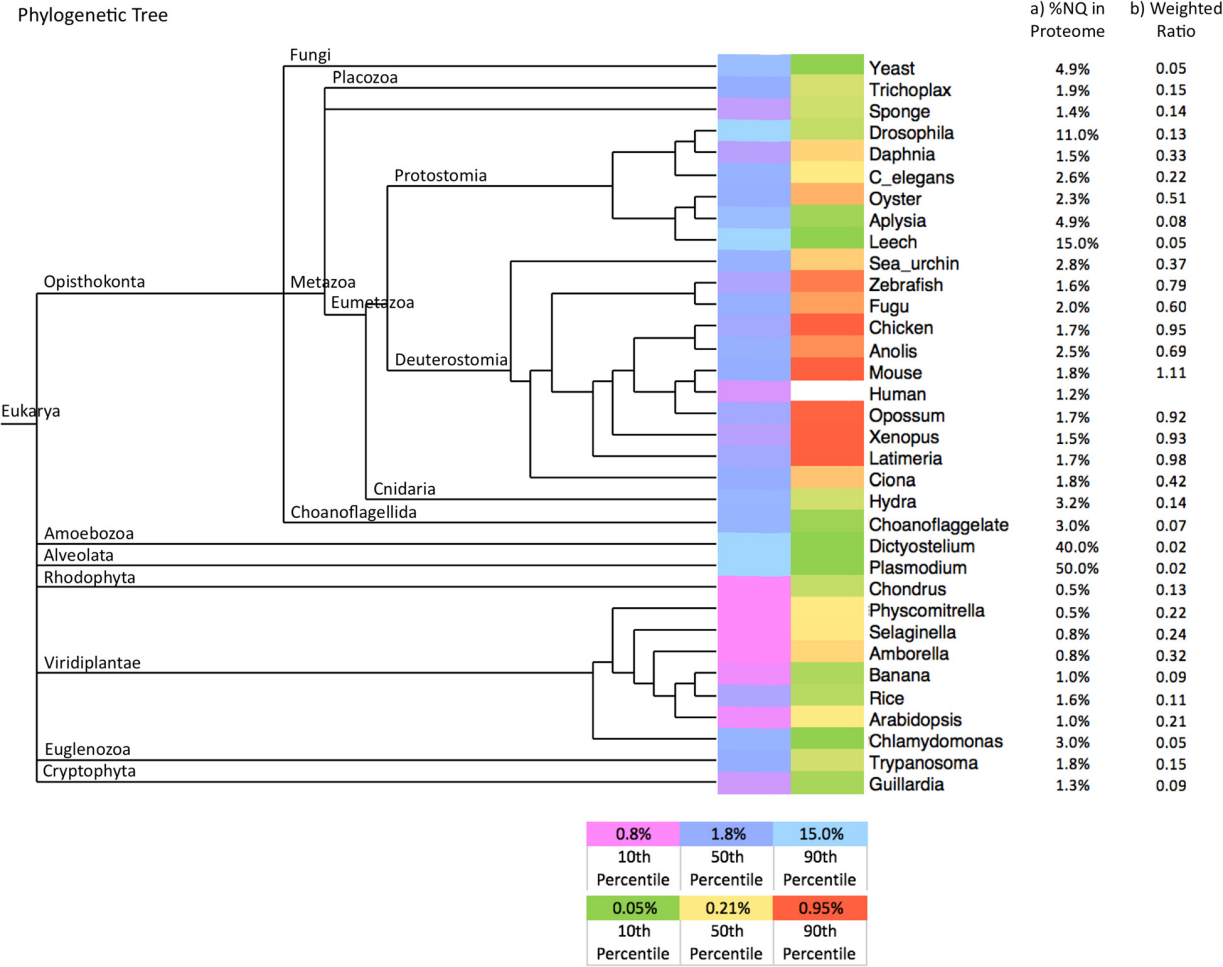
Human yeast-prion-like proteins have limited conservation outside of deuterostomes. The first colour scale (ranging from magenta to cyan) indicates the percentage of N/Q-rich proteins (as determined using the LPS program, see Methods). The second colour scale (from green to red) indicates a weighted ratio that shows the degree of conservation of the human yeast-prion-like proteins. The legends for the colour scales are on the bottom of the figure. The formula for the *weighted ratio* is as follows: $\frac{\left(\mathrm{column}\kern0.49em 8\ /\ \mathrm{column}\ 7\right)\times 100}{\left(1.2/\mathrm{column}\ 1\ \mathrm{percentage}\right)}$. The columns are those from Table 1. The value 1.2 is the percentage of NQPs in the human proteome

Across the eukaryote domain, we see several species with large numbers of NQPs and prion predictions (>10 % NQPs of all proteins), specifically *i.e.*, *Plasmodium, Dictyostelium*, leech and *Drosophila* (Table 1). In *Plasmodium* and *Dictyostelium*, this is due to large amounts of N and/or Q homopeptide runs [41, 42]. In *Drosophila*, there are large numbers of Q-rich regions, particularly associated with proteins functioning in transcription activation/control [28]. Similarly, since the last common ancestor of *Saccharomycetes* yeasts, a large-scale mutational trend for more N homopeptide runs has led to the formation of lots of prion-like proteins in present-day laboratory budding yeast *Saccharomyces cerevisiae*, which are also significantly linked to transcription regulation functions [17]. Human yeast-prion-like proteins analyzed here are similarly linked to transcriptional control (Additional file 1: Table S1). The lowest percentages of yeast-prion-like proteins occur consistently in plants as a clade (Fig. 1).

Human yeast-prion-like proteins have only limited conservation outside of Deuterostomes (Table 1 and Fig. 1; complete list of annotations in Additional file 2: Table S2). In Fig. 1, the conserved numbers of yeast-prion-like proteins are weighted to account for the relative size of the yeast-prion-like protein complements of the other eukaryotes. These statistics indicate that human yeast-prion-like proteins are largely novel since the last common ancestor of Deuterostomes. In general, the yeast-prion-like character of human proteins is found less and less in orthologs at increasing divergences (hypergeometric probabilities, *P =* 9e-06 for conservation outside Tetrapoda, *P =* 7e-07 outside vertebrates, *P =* 4e-09 outside Deuterostomes). Indeed, there are only a handful of orthologous yeast-prion-like proteins between human and yeast (7 yeast prion-like proteins have orthologs in human, 5 of which function in transcriptional control; Table 2, combining NQPs and prion predictions). Only one of these has been shown to have prion-like aggregation in both species, the stress granule assembly component PUB1/TIA1 [43, 44]. This is also the only such yeast-prion-like protein that is genetically linked to a neurodegenerative/ muscular degenerative disease, *i.e.*, Welander distal myopathy, which is caused by heterozygous mutation in PUB1/TIA1 [45]. Generally, yeast-prion-like proteins from *S. cerevisiae* have very low rates of yeast-prion-like orthology across the divergent panel of eukaryotes examined here (0.4-4.9 % of cases; Table 1).

**Table 2:** Orthologous proteins that are yeast-prion-like in both *Saccharomyces cerevisiae* and Human

NQ-rich proteins (NQPs)				
Yeast gene ID	Yeast gene name	Description^a^	Human orthologs^b^	# of species with NQP orthologs^c^
YLL013C	PUF3	Protein of the mitochondrial outer surface; links the Arp2/3 complex with the mitochore during anterograde mitochondrial movement; also functions in mRNA degradation	PUM1 (ENSP00000257075, ENSP00000400141), PUM2 (ENSP00000409905)	16 (30)
YGL237C	HAP2	Subunit of the Hap2p/3p/4p/5p CCAAT-binding complex; complex is heme-activated and glucose-repressed; complex is a transcriptional activator and global regulator of respiratory gene expression	NFYA (ENSP00000229418, ENSP00000345702)	12 (24)
YOR194C	TOA1	TFIIA large subunit; involved in transcriptional activation, acts as antirepressor or as coactivator; required, along with Toa2p, for ribosomal protein gene transcription in vivo	GTF2A1 (ENSP00000409492, ENSP00000452454)	10 (26)
YFL024C	EPL1	Subunit of NuA4, an essential histone H4/H2A acetyltransferase complex; conserved region at N-terminus is essential for interaction with the NPC (nucleosome core particle); required for autophagy	EPC2 (ENSP00000258484)	9 (17)
YNL251C	NRD1	RNA-binding subunit of Nrd1 complex; complex interacts with exosome to mediate 3′-end formation of some mRNAs, snRNAs, snoRNAs, and CUTs; interacts with CTD of RNA pol II large subunit Rpo21p at phosphorylated Ser5 to direct transcription termination of non-polyadenylated transcripts	SCAF4 (ENSP00000382703)	5 (7)
Prion predictions				
Yeast gene ID	Yeast gene name	Description^a^	Human orthologs	# of species with orthologous prion predictions^b^
YLR191W	PAS20	Peroxisomal importomer complex component; integral peroxisomal membrane protein required for docking and translocation of peroxisomal matrix proteins	PEX13 (ENSP00000295030)	15 (19)
YNL016W	PUB1	Poly (A) + RNA-binding protein; abundant mRNP-component protein that binds mRNA and is required for stability of many mRNAs; component of glucose deprivation induced stress granules, involved in P-body-dependent granule assembly; protein abundance increases in response to DNA replication stress. The human ortholog associated with Welandar distal myopathy	TIA1 (ENSP00000404023)	14 (23)
YLL013C	PUF3	*See above*	PUM1 (ENSP00000400141, ENSP00000257075)	13 (30)

^a^These descriptions are adapted from text on the UniProt [37] and SGD databases [49]

^b^The gene names are followed by the Ensembl protein names for the orthologous proteins

^c^Total number of the species listed in Table 1 that contain orthologous prion predictions or NQPs, with the total number of orthologs (regardless of whether they have a prion-like domain or not) in brackets

### Genetic linkage to neurological and neurodegenerative disease

The link between yeast-prion-like proteins and neurological/neurodegenerative diseases has been demonstrated experimentally for several cases, such as FUS and TDP-43 in amyotrophic lateral sclerosis (ALS) [46, 47], and hnRNPA2B1 and hnRNPA1 in ALS and other disease [25]. Also, several neurodegenerative diseases, such as Huntington’s disease, have been shown to be genetically and/or mechanistically linked to proteins that have poly-glutamine (poly-Q) expansions. What is the scale of the role of yeast-prion-like proteins in neurodegenerative diseases, and is it simply a consequence of high expression levels for yeast-prion-like proteins, or a more general linkage to intrinsically disordered proteins? To address these questions, we derived a comprehensive list of yeast-prion-like proteins genetically linked to neurological disease. Lists of genes linked to disease, and more specifically to neurological and neurodegenerative disease, were compiled by data-mining and cross-referencing OMIM and other online resources, as well as the lists of genes encoding yeast-prion-like proteins (as explained in Methods).

**Fig. 2:**
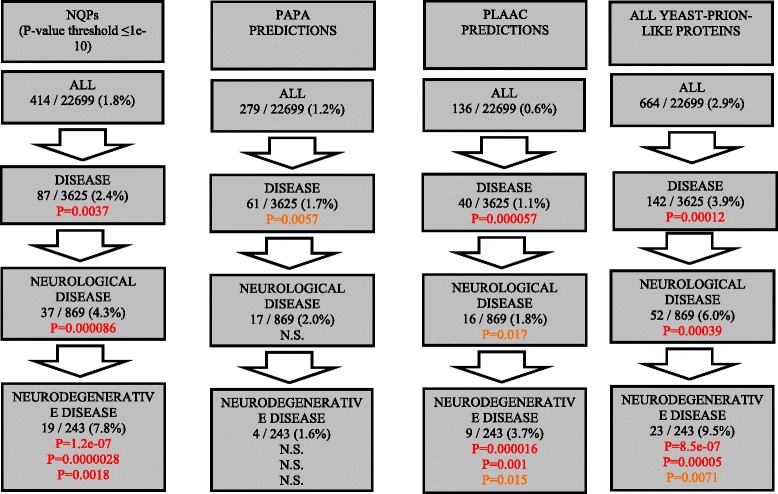
Yeast-prion-like proteins are statistically enriched in human neurological/neurodegenerative diseases. Enrichments of yeast-prion-like proteins are depicted as flow charts. Four sets are shown: NQPs (counted for the LPS P-value threshold ≤1e-10); predictions by the program PAPA [29]; predictions by the program PLAAC [30] (using yeast background settings); the union of all yeast-prion-like proteins from all three sources. These programs were applied as described in Methods. The fraction of all genes, disease-linked genes, neurologically-linked genes and neurodegeneration-linked genes are listed in the boxes. *P* values for hypergeometric tests for enrichments are listed (all of the *P* values are enrichments, no depletions are discovered), relative to the previous set. Cases that might not be significant given a Holm-Bonferroni correction for multiple hypothesis testing are in orange type, otherwise significant cases are in red type. For enrichments in the neurodegenerative set, there are three P-values for comparison to disease-, neurological- and neurodegenerative-linked sets as background populations

For a variety of criteria, there is a significant enrichment of yeast-prion-like proteins in proteins genetically linked to neurological disease, and even more so for the subset that are degenerative (Fig. 2, and Additional file 3: Figure S1). The greatest enrichments are for NQPs annotated using the LPS program, and for prion predictions made using the program PLAAC (Fig. 2). The greatest enrichment for neurodegenerative diseases compared to neurological diseases is for NQPs (*P =* 0.0018, 7.8 % of genes versus 4.3 %). When neurodegeneration-linked genes are compared to disease-linked genes generally the *P* values for NQP enrichment become highly significant (down to *P =* 3.6e-08, Additional file 3: Figure S1).

**Fig. 3:**
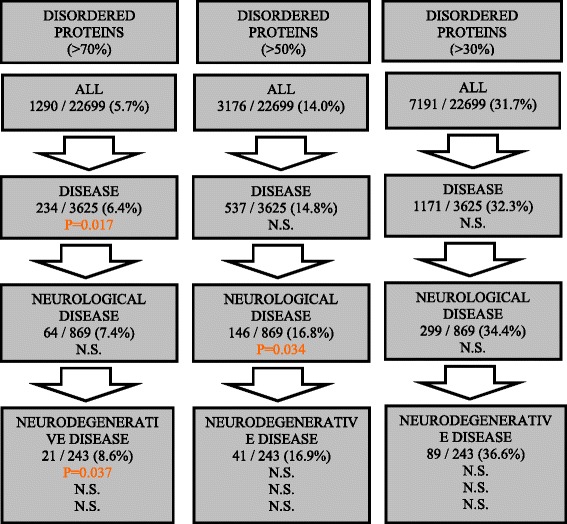
Intrinsically disordered proteins are generally not enriched in neurological/neurodegenerative diseases. These are the same calculations as above for yeast-prion-like proteins, except for three sets of proteins that are intrinsically disordered in the human proteome. Three different thresholds for intrinsic disorder are examined, 30 % of the protein, 50 % and 70 %

The well-characterized prions in *S. cerevisiae* are a subset of proteins with very high intrinsic disorder [11]. Therefore, we checked whether the enrichments that we observe are simply due to enrichments of intrinsically disordered proteins. To do this, we tested for enrichment in neurological diseases for proteins that are not in the sets of yeast-prion-like proteins but which are intrinsically disordered, at three levels of coverage (30 %, 50 % and 70 % disordered; Fig. 3). There are two weak enrichments, *e.g.,* one for proteins >50 % intrinsically disordered, for neurological linkage compared to disease linkage generally (Fig. 3).

The significant enrichments of yeast-prion-like proteins observed for neurological genes relative to disease-linked genes disappears if the neurodegeneration-linked genes are removed from the data. After such a removal, the lowest P-value is for enrichment of NQPs (*P =* 0.09, N.S.). Thus, the effect is largely due to genes linked to neurodegeneration. Also, the enrichments that we observed are not due to genes detected using biased screens for prion-like proteins/genes (Table 3).

**Table 3:** Yeast-prion-like genes genetically linked to neurological diseases in humans^a^

Ensembl gene ID	Name	NQP ?^b^	Prion prediction ?^b^	Neurodegenerative ?^c^	Diseases linked to gene (with OMIM numbers)
Nucleic-acid binding/Transcription factor (PC00171, PC00218)					
ENSG00000104973	MED25, Mediator complex subunit 25	X	--	U	Charcot-Marie-Tooth disease, type 2B2, #605589
ENSG00000112592	TBP, TATA box binding protein	X	X	U	Spinocerebellar ataxia 17, #607136
ENSG00000204842	ATXN2, Ataxin 2	X	--	U	Amyotrophic lateral sclerosis, susceptibility to, 13, #183090;
					Parkinson disease, late-onset, susceptibility to, #168600;
					Spinocerebellar ataxia 2, #183090
ENSG00000085224	ATRX, Alpha thalassemia/mental retardtn. syndrome X-linked	X	X	--	Mental retardation-hypotonic facies syndrome, X-linked, #309580
ENSG00000089280	FUS RNA binding protein	X	X	U	Amyotrophic lateral sclerosis 6, with or without frontotemporal dementia, #608030;
					Tremor, hereditary essential, 4, #614782
ENSG00000100888	CHD8, Chromodomain helicase DNA binding protein 8	X	--	--	Autism, susceptibility to, 18, #615032
ENSG00000160299	PCNT, Pericentrin	X	--	--	Microcephalic osteodysplastic primordial dwarfism, type II, #210720
ENSG00000182944	EWSR1, EWS RNA-binding protein 1	X	X	--	Neuroepithelioma, #612219
ENSG00000111752	PHC1, Polyhomeotic homolog 1	X	X	--	Microcephaly 11, primary, autosomal recessive, #615414
ENSG00000198026	ZNF335, Zinc finger protein 335	X	--	--	Microcephaly, #615095
ENSG00000052850	ALX4, ALX homeobox 4	+	--	--	Craniosynostosis 5, #615529
ENSG00000066427	ATXN3, Ataxin 3	X	--	U	Machado-Joseph disease, #109150
ENSG00000140521	POLG, Polymerase (DNA directed), ©	X	--	U	Mitochondrial recessive ataxia syndrome (includes SANDO and SCAE), #607459
ENSG00000156876	SASS6, SAS-6 centriolar assembly protein	+	--	--	Microcephaly 14, primary, autosomal recessive, #616402
ENSG00000169083	AR, Androgen receptor	X	--	U	Spinal and bulbar muscular atrophy of Kennedy, #313200
Cytoskeletal protein (PC00085)					
ENSG00000066279	ASPM, abnormal spindle microtubule assembly	X	X	--	Microcephaly 5, #608716
ENSG00000131018	SYNE1, Spectrin repeat containing nuclear envelope 1	X	--	U	Emery-Dreifuss muscular dystrophy 4, autosomal dominant, #612998;
					Spinocerebellar ataxia, autosomal recessive 8, #610743
ENSG00000151914	DST, Dystonin	X	--	--	Neuropathy, hereditary sensory and autonomic, type VI, #614653
ENSG00000008056	SYN1, synapsin I	X	--	--	X-linked epilepsy, #300491
ENSG00000133454	MYO18B, Myosin XVIIIB	X	--	--	Klippel-Feil syndrome 4, autosomal recessive, with myopathy, #616549
ENSG00000166813	KIF7, Kinesin family member 7	X	--	--	Joubert syndrome, etc., #200990
ENSG00000178209	PLEC, Plectin	X	--	CM	various muscular dystrophy, #226670, #613723
ENSG00000277586	NEFL, Neurofilament light polypeptide	--	X	U	Charcot-Marie-Tooth disease, #607734, #607684
ENSG00000054654	SYNE2, Spectrin repeat containing, nuclear envelope 2	+	--	CM	Emery-Dreifuss muscular dystrophy 5, #612999
ENSG00000100345	MYH9, Myosin, heavy chain 9, non-muscle	+	--	--	Macrothrombocytopenia and progressive sensorineural deafness, #600208
ENSG00000138778	CENPE, Centromere protein E, 312 kDa	+	--	--	Microcephaly 13, primary, autosomal recessive, #616051
ENSG00000198947	DMD, Dystrophin	+	--	CM	Becker muscular dystrophy, #300376; Duchenne muscular dystrophy, #310200
Transporter (PC00227)					
ENSG00000157388	CACNA1D, Ca channel, voltage-depdt., L type, 〈 1D subunit	--	X	--	Primary aldosteronism, #615474
					Sinoatrial node dysfunction and deafness, #614896
ENSG00000198734	F5, Coagulation factor V (proaccelerin, labile factor)	--	X	--	Suscept. to stroke, #601367
ENSG00000007314	SCN4A, Na channel, voltage gated, type IV 〈 subunit	--	X	--	Hyperkalemic periodic paralysis, type 2, #170500, #613345;
					Myotonia congenita, atypical, acetazolamide-responsive, #608390; Paramyotonia congenita, #168300
ENSG00000036828	CASR, Calcium-sensing receptor	X	--	--	Idiopathic generalized epilepsy, #612899
ENSG00000141837	CACNA1A, Ca channel, voltage-depdt., P/Q type, 〈 1A subunit	X	--	U	Spinocerebellar ataxia 6, #183086;
					Migraine, familial hemiplegic, 1, with progressive cerebellar ataxia, #141500;
					Episodic ataxia, type 2, #108500;
ENSG00000164588	HCN1, Hyperpolariztn. activated cyclic-nucleotide-gated K channel 1	X	--	--	Epileptic encephalopathy, early infantile, 24, #615871
Others					
ENSG00000116001	TIA1, TIA1 cytotoxic granule-associated RNA binding protein	+	X	CM	Welander distal myopathy, #604454
ENSG00000157212	PAXIP1, PAX interacting protein 1	X	X	CN	Alzheimer disease, susceptibility to, #104300
ENSG00000162928	PEX13, Peroxisomal biogenesis factor 13	--	X	--	Peroxisome biogenesis disorder 11A (Zellweger), #614883
ENSG00000197386	HTT, Huntingtin	X	X	U	Huntington disease, #143100
ENSG00000204120	GIGYF2, GRB10 interacting GYF protein 2	X	--	U	Parkinson disease 11, #607688
ENSG00000103995	CEP152, Centrosomal protein 152 kDa	X	--	--	Microcephaly 9, primary, autosomal recessive, #614852
ENSG00000114354	TFG, TRK-fused gene	X	X	U	Hereditary motor and sensory neuropathy, Okinawa type, #604484;
					Spastic paraplegia 57, autosomal recessive, #615658
ENSG00000120948	TARDBP, TAR DNA binding protein	--	X	U	Amyotrophic lateral sclerosis 10, with or without FTD, #612069;
					Frontotemporal lobar degeneration, TARDBP-related, #612069
ENSG00000122566	HNRNPA2B1, Heterogen. nuclear ribonucleoprotein A2B1^d^	--	X	CN^d^	Inclusion body myopathy with early-onset Paget disease, #615422
ENSG00000135486	HNRNPA1, Heterogeneous nuclear ribonucleoprotein A1^d^	--	X	U^d^	Amyotrophic lateral sclerosis 20, #615426;
					Inclusion body myopathy wtih early-onset Paget disease without frontotemporal dementia 3, #615424
ENSG00000136352	NKX2-1, NK2 homeobox 1	X	--	--	Chorea, hereditary benign, #118700;
					Choreoathetosis, hypothyroidism, and neonatal respiratory distress, #610978
ENSG00000145868	FBXO38, F-box protein 38	X	--	U	Neuronopathy, distal hereditary motor, type IID, #615575
ENSG00000152795	HNRNPDL, Heterogeneous nuclear ribonucleoprotein D-like	--	X	CM	Limb-girdle muscular dystrophy, type 1G, #609115
ENSG00000154118	JPH3, Junctophilin 3	--	X	CN	Huntington disease-like 2, #606438
ENSG00000168000	BSCL2, Berardinelli-Seip congenital lipodystrophy 2 (seipin)	--	X	U	Encephalopathy, progressive, with or without lipodystrophy, #615924;
					Neuropathy, distal hereditary motor, type VA, #600794;
					Silver spastic paraplegia syndrome, #270685
ENSG00000186472	PCLO, Piccolo presynaptic cytomatrix protein	X	--	U	Pontocerebellar hypoplasia, type 3, #608027
ENSG00000269335	IKBKG, Inhibitor of | light polypeptide B-cell gene enhancer, kinase ©	X	--	--	Incontinentia pigmenti, #308300
ENSG00000027075	PRKCH, Protein kinase C, eta	--	X	--	Cerebral infarction, susceptibility to, #601367
ENSG00000060237	WNK1, WNK lysine deficient protein kinase 1	X	X	U	Neuropathy, hereditary sensory and autonomic, type II, #201300
ENSG00000074047	GLI2, GLI family zinc finger 2	+	--	--	Holoprosencephaly-9, #610829
ENSG00000111676	ATN1, Atrophin 1	X	X	U	Dentatorubro-pallidoluysian atrophy, #125370
ENSG00000124788	ATXN1, Ataxin 1	X	X	U	Spinocerebellar ataxia 1, #164400
ENSG00000127838	PNKD, Paroxysmal nonkinesigenic dyskinesia	--	X	--	Paroxysmal nonkinesigenic dyskinesia, #118800
ENSG00000148356	LRSAM1, Leucine rich repeat and sterile motif containing 1	X	--	U	Charcot-Marie-Tooth disease, axonal, type 2P, #614436
ENSG00000163635	ATXN7, Ataxin7	X	--	U	Spinocerebellar ataxia 7, #164500
ENSG00000164342	TLR3, Toll-like receptor 3	+	--	--	Herpes simplex encephalitis, susceptibility to, 2 #613002
ENSG00000188021	UBQLN2, Ubiquilin 2	+	--	U	Amyotrophic lateral sclerosis 15, with or without frontotemporal dementia, #300857

^a^The genes are grouped according to the three most common PANTHER protein classes [48]. In making these listings, the four organisms with very high rates of yeast-prion-like proteins (see Table 1) are not considered

^b^NQPs are N/Q-rich proteins as defined. In the ‘NQP?’ column, genes which encode an NQP with LPS *P*-value <1e-10 are labelled with an ‘X’ , those with *P*-value otherwise <1e-08 are labelled with a ‘+’ sign. If the gene has an algorithmic prion prediction, it is labelled with an ‘X’ in the ‘Prion Prediction?’ column

^c^Genes that are in the UniProt neurodegenerative list are labelled ‘U’. To other labels arise from curation of the scientific literature: those that were determined as neurodegeneration-linked genes are labelled ‘CN’ , whereas those that are specifically linked to muscular degeneration are labelled ‘CM’

^d^These two were identified as linked to neurodegenerative illnesses after a biased screen for proteins with prion-like domains. However, they have previously identified links to neurological illnesses. Removal of these two cases found in such screens (hnRNPA2B1 and hnRNPA1, which are linked to ALS and other muscular degeneration [25]) does not affect the calculations. Also, these two genes have previously discovered genetic linkages to neurodegenerative disorders [50, 51]

Given the observed genetic linkages, is there perhaps significant tissue-specific expression of yeast-prion-like proteins in the central nervous system or in muscle? Generally for these three tissues, there is typical representation of yeast-prion-like proteins, with the exception of a possible significant excess of NQPs that are tissue-specific to skeletal muscle (Table 4). This excess may thus be a correlate of the incidence of muscular degenerative diseases that are caused by yeast-prion-like proteins in muscle cells, since higher expression may be required to maintain a supply of protein for aggregate formation, as muscle cells undergo cell division.

**Table 4:** Tissue-specifically expressed subsets of genes in relevant human tissues^a^

Protein sets tested and expression sets used (columns) Tissue types (rows)	NQ-rich proteins^b^		Prion predictions by PLAAC^b^		Prion predictions by PAPA^b^		DISORDERED PROTEINS (>70 % set)^b^	
	GXA Illumina body map expression library	Specond	GXA Illumina body map expression library	Specond	GXA Illumina body map expression library	Specond	GXA Illumina body map expression library	Specond
Brain	38/1966 (1.9 %, N.S.)	9/503 (1.8 %, N.S.)	10/1966 (0.5 %, N.S.)	3/503 (0.6 %, N.S.)	25/1966 (1.3 %, N.S.)	8/503 (1.6 %, N.S.)	127/1966 (6.5 %, N.S.)	42/503 (8.4 %, N.S.)
Spinal Cord	N/A	3/262 (1.1 %, N.S.)	N/A	2/262 (1.1 %, N.S.)	N/A	3/262 (1.1 %, N.S.)	N/A	**31/262** **(11.8 %,** ***P*** **=** **0.001)**
Skeletal Muscle	**25/** **753 (3.3 %,** ***P*** **=** **0.015)**	5/270 (1.9 %, N.S.)	6/753 (0.8 %, N.S.)	3/270 (1.1 %, N.S.)	4/753 (0.5 %, N.S.)	4/270 (1.5 %, N.S.)	52/752 (7.0 %, N.S.)	19/270 (7.0 %, N.S.)

^a^Enrichments are in bold type. For each is listed the fraction, with the percentage and hypergeometric *P*-value in brackets (N.S. = not significant)

^b^For NQ-rich proteins, the LPS threshold used is 1e-10 (overall fraction is 414/22699, 1.8 %). The PLAAC and PAPA predictions use default values (overall fractions: 136 / 22699 (0.6 %) PLAAC; 279 / 22699 (1.2 %) PAPA). In all cases, human protein composition is used for these annotations. The disordered proteins are annotated as described in Methods

The small numbers of human yeast-prion-like proteins that have yeast-prion-like orthology outside deuterostomes are typically likely to be involved in neurological disease. For example, for NQPs ~10 % (17/177) of such proteins are linked to neurological disease versus ~9 % (38/414) of NQPs overall. Similarly, ~6 % (10/177) are linked to neurodegenerative disease versus ~5 % (21/414) of NQPs overall. (In calculating these conservation statistics, the four species with very high percentages of yeast-prion-like proteins, labelled in Table 1, are not included).

We have compiled a complete list of the yeast-prion-like proteins that are linked to neurological diseases in humans (Table 3). For this list we curated the scientific literature to pick out any further cases that are linked to neurodegeneration or muscular degeneration (Table 3). Genes linked to neurodegeneration/muscular degeneration dominate the list, comprising more than half (32/60) of cases. The genes are grouped according to the three largest PANTHER protein classes [48] (which are incidentally non-overlapping sets). These are: ‘Nucleic-acid binding/transcription factor’ , ‘Cytoskeletal protein’ and ‘Transporter’ , (with ‘Others’ listed at the end of the table). Cytoskeletal proteins are significantly overrepresented (Additional file 4: Table S3). Similarly, there is a significant enrichment of proteins associated with the GO ‘microtubule-based process’ category (Additional file 1: Table S1). In examining this table, we also noted that several yeast-prion-like genes are linked to microcephaly (7 cases), so we checked whether these are significantly linked, and this is indeed the case (7/36, 19 % of all microcephaly-linked genes in the OMIM database, hypergeometric *P*-value = 0.009). This prompts the hypothesis that protein aggregation/amyloidogenesis may be a prominent factor in the aetiology of some of these disorders.

### Overlap between annotation methods

We assessed the overlap between the different annotation methods for yeast-prion-like proteins in the human proteome (Additional file 5: Figure S2). We observe a core set of 101 proteins that are annotated as yeast-prion-like by all of the methods (listed at the end of Additional file 2: Table S2). Surprisingly, different annotation methods only have limited overlap. Particularly, 26 % of LPS annotations (using the binomial *P*-value threshold of 1e-10), are predicted to have prion domains by PAPA or PLAAC. Conversely, 89 % of PLAAC prions predictions (using yeast background composition) and all of the PAPA predictions are annotated as prions by other methods. However, PAPA predictions do not yield a significant enrichment in relation to human neurological diseases (Fig. 2). These results are explicable if one considers that PLAAC and PAPA are trained on sequences that form prions *in vivo* in *S. cerevisia*e cells. Thus, prion-like domains in humans quite possibly have a different amino-acid composition, so that bias for Q and N is supplemented with different amino acids to those seen in *S. cerevisiae* prion-forming domains.

## Conclusions

The yeast-prion-like domains in human proteins are largely novel since the last common ancestor of Deuterostomes, although any with yeast-prion-like orthologs outside Deuterostomes have a similar involvement in neurological/neurodegenerative diseases. The yeast-prion-like proteins genetically linked to neurodegenerative illnesses are a large enough set to cause substantial enrichments, by some criteria highly statistically significant. They also dominate the list of genetic linkages of yeast-prion-like proteins for neurological illnesses generally. Our results motivate further probing of the nature of the link between yeast-prion-like proteins and neurological pathomechanisms in humans. Our results also indicate that further experimental refinement of the compositional criteria in humans for forming yeast-prion-like proteins is required.

## Reviewers’ comments

### Reviewer’s report 1: István Simon (Institute of Enzymology, Budapest, Hungary)

In this manuscript, the authors consider proteins as yeast-protein-like ones if it has a domain with pronounced N/Q-rich regions, determined by the program LPS or a prion domain, predicted by the program PAPA or PLAAC. Prion is the prototype of plaque forming proteins and prions also known as partly unstructured protein. Plaque formation is very common in many neurological diseases and unstructured proteins, is known as less conservatives due to the lack of structural constrain.

Therefore, although it is a correct work, but the authors should present some less obvious conclusions than the lack of conservation and the link to neurological diseases of the yeast-protein-like, presented in this version.

Authors’ Response: *We disagree that these two results are obvious; these observations have not been reported before. It was not previously obvious how deeply conserved human yeast-prion-like proteins are, also the extent of the linkage of yeast-prion-like proteins to neurological illnesses has not been previously quantified. Also there are a lot of other results in the paper. We present statistics of yeast-prion-like proteins for a diverse panel of eukaryotes, and analyse the proteins that are yeast-prion-like in both human and yeast. Furthermore, we show that the linkage of yeast-prion-like proteins to neurological illnesses is not due to expression levels or to a more general trend for intrinsically disordered proteins. Also, we found that linkage to neurological disease is not related to depth of conservation. The compilation of neurologically relevant yeast-prion-like proteins is also a useful resource for experimentalists.*

*We have added some further results for the important protein classes of neurological yeast-prion-like proteins, and analysis of the overlap between the annotations of yeast-prion-like proteins made using three different methods. Also, we have revised the title of the paper to the more general: “The evolutionary scope and neurological disease linkage of yeast-prion-like proteins in humans”.*

### Reviewer’s report 2: Alexander Schleiffer (CSF Vienna Biocenter, Austria)

An and Harrison demonstrate an interesting statistically significant link of yeast-prion like proteins to human neurological diseases, particularly neurodegenerative diseases.

Three distinct programs were applied to determine a set of prion-like proteins (LPS, PAPA and PLAAC). Judging from the information in Table 2, the overlap of these predictions is surprisingly low, although they are all based on amino acid composition. The authors should specify the size of the intersections of these algorithms for the human proteome.

Authors’ Response: *We have added a section discussing this (at the end of Results & Discussion), and it is mentioned in the abstract and conclusions sections.*

Minor Issues:

Page 8, first paragraph: 7 proteins are reported to be conserved between yeast and human, however 9 proteins are listed in Tables 1 and 2.

Authors’ Response: *This has been corrected/clarified.*

Figure 1: can be easily removed, since it does not provide any additional information than that already presented in Table 1 and the color code is difficult to comprehend in this resolution.

Authors’ Response: *We disagree that the Figure is superfluous; it illustrates a calculation graphically that is not included in Table**1**, and it shows the organismal phylogeny for the data that we analyzed. The colour heatmaps have been made larger, and we have made sure that the individual Figure file is high resolution as specified in the Author Guidelines (in the generated PDF it is depicted in low resolution, but there is a link to the original figure file in the upper right-hand corner of the PDF page).*

Figure 2: the last set is supposed to be the union of the first three prediction programs, but the number of all yeast-prion like proteins is higher than the sum of the single predictions. The authors should explain.

Authors’ Response: *We had used the numbers using a higher LPS bias P-value threshold (1e-08). We have corrected it. The numbers do not add up exactly because there is substantial overlap in the assignments made using the three methods.*

Table 2: in the human orthologs column isoforms and paralogs are listed. Specifying human gene names would avoid confusions. It can also be informative, if the total number of species with orthologs is added. For instance, in the case of genes with only a few NQP orthologs, such as NRD1, it is not clear whether they are less conserved or simply lost the NQP region.

Authors’ Response: *We have added this information to the table.*

Table 3 lists all yeast prion-like genes that are linked to a human disease. Since the emphasis is on the disease, grouping according to conservation is less meaningful than according to disease or biological process. For example, a prominent group consists of genes involved in cytoskeleton organization and microtubule dynamics (such as ASPM, CENPE, CEP152, ATXN3, ATXN7, SASS6, DST, MYH9, SYNE1, SYNE2, PLEC, and NEFL); maybe in this case PANTHER (http://www.pantherdb.org/) can be of use.

Authors’ Response: *There are significant enrichments of cytoskeletal proteins and sodium and calcium-gated channels (Additional File). The largest three PANTHER protein classes are given in Table**3**(these three classes are non-overlapping for this data set).*

A complete list of all predicted NPQ proteins in human (including gene names) as supplementary information is valuable for the readers.

Authors’ Response: *We have made the Additional file.*

## Abbreviations

ALS, amyotrophic lateral sclerosis; GO, Gene Ontology; N, asparagine; NCBI, National Center for Biotechnology Information; NINDS, National Institute for Neurological Diseases and Stroke; NQP, asparagine/glutamine-rich protein; OMIM, Online Mendelian Inheritance in Man; poly-Q, poly-glutamine; Q, glutamine

## Additional files

Additional file 1: Table S1. Gene Ontology (GO) process category enrichments for the NQP and prion prediction data sets from human (the same sets that are analyzed in Tables 1, 2, 3 and 4). These are derived using the website GOrilla [52]. (TXT 3 kb) Additional file 2: Table S2. The lists of human proteins/genes with prion-like domains are listed for the programs applied in the paper: LPS, PLAAC and PAPA. (TXT 167 kb) Additional file 3: Figure S1. Enrichments in NQPs, using other binomial *P*-value thresholds for the LPS program [11, 27, 28]. The flowcharts are laid out as in Figs. 2 and 3. (DOC 64 kb) Additional file 4: Table S3. Significant PANTHER protein class enrichments for the NQP and prion prediction data sets from human (the same sets that are analyzed in Tables 1, 2, 3 and 4). These are derived using the PANTHER website [48]. (TXT 786 bytes) Additional file 5: Figure S2. Venn diagrams showing the overlap of the annotations using LPS, PAPA and PLAAC. The Venn diagrams are made using a specialized website [53]. (DOCX 261 kb)
