# Supplementary material for: The evolutionary scope and neurological disease linkage of yeast-prion-like proteins in humans
Source: Biol Direct. 2016 Jul 26;11:32. doi: 10.1186/s13062-016-0134-5 (PMC4960796; doi:10.1186/s13062-016-0134-5)
Supplement: Additional file 3: Figure S1. — Enrichments in NQPs, using other binomial P-value thresholds for the LPS program [11, 27, 28]. The flowcharts are laid out as in Figs. 2 and 3. (DOC 64 kb) [file 13062_2016_134_MOESM3_ESM.doc]

ALL

305 / 22699 (1.3%)

DISEASE

67 / 3625 (1.8%)

P=0.0035

NEUROLOGICAL DISEASE

30 / 869 (3.5%)

P=0.00013

NEURODEGENERATIVE DISEASE

17 / 243 (7.0%)

P=3.6e-08

P=0.000001

P=0.00073

414 / 22699 (1.8%)

414 / 22699 (1.8%)

414 / 22699 (1.8%)

NQPs

(threshold 1e-12)

ALL

646 / 22699 (3.8%)

DISEASE

126 / 3625 (3.5%)

N.S.

NEUROLOGICAL DISEASE

48 / 869 (5.5%)

P=0.00022

NEURODEGENERATIVE DISEASE

20 / 243 (8.2%)

P=0.000023

P=0.00021

P=0.025

414 / 22699 (1.8%)

414 / 22699 (1.8%)

414 / 22699 (1.8%)

NQPs

(threshold 1e-08)
