# Supplementary material for: The evolutionary scope and neurological disease linkage of yeast-prion-like proteins in humans
Source: Biol Direct. 2016 Jul 26;11:32. doi: 10.1186/s13062-016-0134-5 (PMC4960796; doi:10.1186/s13062-016-0134-5)
Supplement: Additional file 5: Figure S2. — Venn diagrams showing the overlap of the annotations using LPS, PAPA and PLAAC. The Venn diagrams are made using a specialized website [53]. (DOCX 261 kb) [file 13062_2016_134_MOESM5_ESM.docx]

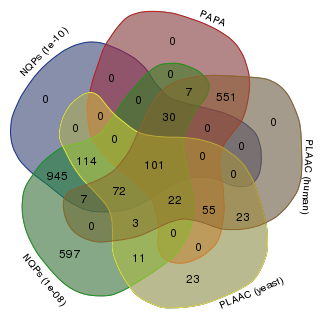

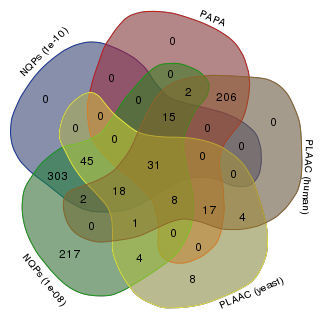


PROTEINS GENES

| **Set** | **# of proteins** | **# of genes** |
| --- | --- | --- |
| NQPs (threshold 1e-08) | 1909 | 646 |
| NQPs (threshold 1e-10) | 1269 | 414 |
| PAPA | 766 | 276 |
| PLAAC (yeast background) | 424 | 134 |
| PLAAC (human background) | 871 | 304 |
